# Increasing indices of frailty in aged female mice are associated with impaired skeletal muscle resilience to downhill running stress

**DOI:** 10.1007/s11357-025-01856-7

**Published:** 2025-09-11

**Authors:** Grant R. Laskin, Laís R. Perazza, Ted G. Graber, Baylah R. Mazonson, Yuhoung J. Kim, Laura J. Verdi, LaDora V. Thompson

**Affiliations:** 1 Department of Physical Therapy, Boston University, Boston, MA USA; 2 Department of Physical Therapy, East Carolina University, Greenville, NC USA

**Keywords:** Muscle contractility, Mitochondrial dysfunction, Stress resilience, Frailty

## Abstract

Frailty is a clinical syndrome marked by diminished physiological reserve and function. While skeletal muscle dysfunction is central to frailty, most preclinical models focus on basal function and place less emphasis on physiological stress responses. Here, we examined the influence of increased indices of frailty on skeletal muscle resistance and resilience using a physiologically relevant model of downhill running stress. Aged female C57BL/6JN mice (*n* = 47; > 17 months) were stratified into Low (≤ 1 frailty markers) or High (≥ 2 frailty markers) groups based on their number of positive frailty markers. Mice were subsequently randomized to undergo two bouts of downhill running or remain cage sedentary. Twenty-four hours later, contractile function was assessed ex vivo in the extensor digitorum longus (EDL) and soleus muscles. RNA was extracted from the gastrocnemius of randomly selected samples and analyzed by RNA sequencing. Despite comparable specific tetanic force, High frailty marked mice exhibited greater fatiguability and impaired recovery kinetics in the EDL following running stress. RNA sequencing revealed divergent transcriptional signatures between mice in Low and High frailty marked groups in response to running, including upregulation of mitochondrial bioenergetic and complex assembly pathways in Low group mice and downregulation in High group mice. These data demonstrate that downhill running stress unmasks latent impairments with frailty in skeletal muscle, and that mitochondrial dysfunction and/or redox imbalance may be potential contributors to reduced muscle resilience. Overall, our results emphasize the importance of incorporating physiological stress paradigms to uncover frailty-associated muscle impairments that are not apparent under basal conditions.

**Graphical Abstract:**

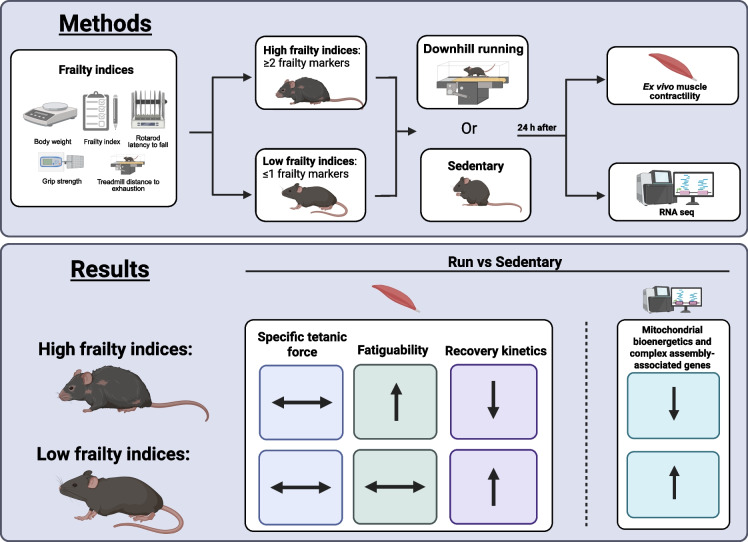

**Supplementary Information:**

The online version contains supplementary material available at 10.1007/s11357-025-01856-7.

## Introduction

Recent progress in clinical and health-related research has substantially increased global life expectancy (e.g., chronological age). Individuals are living longer now than any other time in human history, and a recent World Health Organization (WHO) report approximates that by 2050 the number of people over the age of 60 will exceed 2 billion [1]. However, this extension in lifespan is accompanied by a severe burden. Distinct from chronological aging, frailty is a clinical syndrome that has coordinately increased in prevalence parallel to the increases in lifespan. Frailty is characterized by reduced physiological reserve and function, and is attributed to disability, morbidity, and hospitalization in older adults [2, 3]. A defining feature of frailty is not merely diminished basal function, but rather the inability to maintain (e.g., resistance) or recover functionality (e.g., resilience) following stress [4, 5]. Despite this, most preclinical models assess baseline characteristics and rarely examine dynamic responses to physiological stress.

Accumulating evidence suggests that several frailty-related physiological deficits often remain latent and only emerge under stress. To illustrate, skeletal muscle dysfunction is a hallmark of frailty and a major contributor to loss of mobility and independence in aging populations [2, 3, 6, 7]. While age-associated reductions in muscle mass (e.g., sarcopenia) partially account for some functional losses, several impairments can remain undetected until the muscle is exposed to a stressor. For example, chronic contractile activity enhances muscle performance over time in young rats but diminishes it in older counterparts, despite similar baseline isometric forces [8]. Additionally, the skeletal muscle of older humans and rodents exhibits impaired recovery and regeneration following a damaging stimulus [9–12]. Although evaluating frailty under stress conditions is essential, direct comparisons of skeletal muscle responses to physiologically relevant stress in individuals or rodents with differing degrees of frailty remain limited. Moreover, many existing stress models employ supraphysiological insults (e.g., cardiotoxin) [13] which may not be representative of stress encountered in daily life. Thus, functional assessments incorporating physiologically relevant stressors are required to uncover the full extent of muscular impairment in frailty.

The purpose of this study was to define the extent to which increased indices of frailty influences resilience and resistance to physical stress in skeletal muscle. To accomplish this, we stratified aged female mice into Low or High groups based on their number of positive frailty markers, then subjected them to a downhill running protocol followed by ex vivo whole muscle contractility. Downhill running is a well-established physiological stressor that imposes eccentric mechanical strain on the lower limb musculature, modeling unaccustomed activity in humans. We show that an increased degree of frailty compromises both fatigue resistance and recovery kinetics following downhill running, and that these impairments are accompanied by alterations to mitochondrial complex regulatory gene expression. Our findings provide critical insight into frailty-associated impairments in muscle stress responses and elucidate potential molecular pathways contributing to diminished resilience in frail skeletal muscle.

## Methods

### Animal and ethical approval

Female C57BL/6JN mice (*n* = 47) were obtained from the National Institute on Aging (NIA) rodent colony and selected to be > 17 months of age at study entry, corresponding to the reported onset of frailty in this strain [14]. Female mice were selected for translational relevance as female humans and rodents are at higher risk of frailty [15]. Mice were housed in a temperature-controlled (25 °C) facility on a 12-h light/dark cycle at the Boston University vivarium, with ad libitum access to standard rodent chow and water. Tissues were collected under anesthesia (10 mg/mL ketamine and 1 mg/mL xylazine in PBS), and euthanasia was performed by exsanguination following cardiac excision. All procedures were approved by the Boston University Institutional Animal Care and Use Committee (IACUC; Protocol #202,000,060).

### Assessment of frailty indices and experimental design

**Fig. 1:**
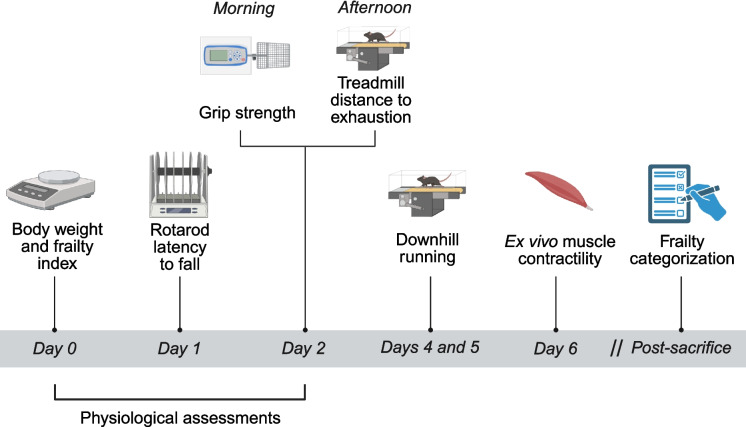
Experimental timeline

The timeline of experimental procedures is provided in Fig. 1. Upon study entry, aged female mice were assessed for the extent of frailty using established parameters [14, 16]. Briefly, mice were evaluated on five criteria: three physical performance tests (treadmill distance to exhaustion, grip strength, and Rotarod latency to fall), body weight, and frailty index (FI). Assessments were performed at the same time of day to minimize circadian variability and spaced apart accordingly to minimize performance interference between tests. A single evaluator conducted all assessments for each parameter. Mice received a positive frailty marker if they fell below the 20th percentile of the cohort for physical tests, or above the 80th percentile for body weight or FI.

Mice were then randomly assigned to sedentary control (*n* = 26) or downhill running (*n* = 21) groups. Running groups underwent two consecutive days of downhill running, while sedentary mice remained in their cages. Mice were anesthetized 24 h after the last running bout, and the soleus and extensor digitorum longus (EDL) muscles were extracted for ex vivo contractility analyses. Remaining tissues were snap-frozen in liquid nitrogen and stored at –80 °C. Following sacrifice, frailty markers were totaled for each mouse and animals were retrospectively assigned to either a High frailty marked group (Control: *n* = 7; Run: *n* = 5) if they exhibited ≥ 2 positive markers, or a Low frailty marked group (Control: *n* = 19; Run: *n* = 16) if they exhibited ≤ 1 marker. This stratification enabled assessment of the impact of increasing indices of frailty on muscle dysfunction.

### Treadmill distance to exhaustion

Distance to exhaustion was assessed using a six-lane motorized treadmill (Exer 3/6 Treadmill; Columbus Instruments, Columbus, OH). Mice performed a 5 min warm-up at 5 m/min on a level deck, after which the speed increased by 1 m/min every minute. A shock grid at the end of each lane provided motivation to continue running. Exhaustion was defined as remaining on the shock grid for three consecutive seconds.

### Grip strength

Grip strength was measured using a grip strength meter (Grip Strength Test P/N 760483; Coulbourn Instruments, Whitehall, PA). Mice were gently lowered onto the grid to allow both forelimbs and hindlimbs to grasp the bars. The tail was then steadily pulled backward, keeping the torso horizontal by holding the base of the tail between the thumb and forefinger. The trial ended when the mouse released its grip, and maximal force was recorded. Five trials were performed with a 10 min rest between each trial. The highest and lowest values were excluded, and the mean of the remaining three trials was used for analysis.

### Rotarod latency to fall

Latency to fall was assessed using a rotarod apparatus (PanLab Letica Rota-Rod L/S; Catalonia, Spain). Mice were acclimated for 1 min at 4 rpm before the speed was increased by 1 rpm every 8 s, reaching a maximum of 40 rpm over 5 min. Testing continued until the mouse could no longer maintain its position and fell from the rod. Each mouse underwent three trials with a 10 min rest between trials. Latency to fall was calculated as the average of the three trials.

### Frailty index

The frailty index (FI) was assessed at baseline using a deficit accumulation scale. An adapted version of the previously described 31-item scale [17] was used which excluded forelimb grip strength, body temperature, and body weight, resulting in 28 evaluated health deficits. Deficits included vestibular disturbance, alopecia, dermatitis, coat condition, vision and hearing loss, among others, and were assessed blindly by a single rater. Each deficit was scored as 0 (no deficit), 0.5 (mild deficit), or 1 (severe deficit). The FI score for each mouse was calculated by summing the individual deficit scores and dividing by the total number of assessed deficits (e.g., 28).

### Downhill running stress

Downhill running was performed using a six-lane motorized treadmill set to a 22 decline as described [18]. The protocol began with a 6 min warm-up period beginning at 5 m/min with speed gradually increased to 13 m/min. After a 3 min rest, mice completed 10 stages consisting of a 1 min acceleration period followed by a 6 min run at 13 m/min and a subsequent 3 min rest. The mice were manually prodded to encourage running. If the mouse refused to run despite encouragement, they were provided a 3 min rest period before reinitiating the run.

### Ex vivo whole muscle contractility

All procedures were performed using methods similar to those previously used by our laboratory [19]. Animals were anaesthetized by intraperitoneal injection of ketamine/xylazine and maintained on a heat pad. The soleus and EDL muscles were excised and immediately transferred to a tissue bath filled with continuously oxygenated Krebs–Ringer buffer (115 mM NaCl, 5.9 mM KCl, 1.2 mM MgCl_2_, 1.2 mM NaH_2_PO_4_, 1.2 mM Na_2_SO_4_, 2.5 mM CaCl_2_, 25 mM NaHCO_3_, and 10 mM dextrose), maintained at 25°C. Each muscle was tied at both tendons with a 4–0 braided silk suture and mounted between a force transducer and a static clamp between two platinum electrodes. Muscle contractility was measured using a dual-bath physiology system (Aurora Scientific, Aurora, Ontario, Canada) consisting of two force transducers (0.5 N, Model 300B), two stimulators (Model 701B), one Dual Lever A/D Interface (Model 604B), one Dual System Signal Interface, customized Aurora software Dynamic Muscle Control (version 4.1.4.6), Dynamic Muscle Analysis (version 3.2), and a temperature control with water bath unit (Model 912; Polyscience, Niles, IL). Stimulation was delivered in biphase modality at an output of 1000 mA and 30 V. Contractile measures were performed at optimal length (*L*_0_), determined by incrementally increasing muscle length until maximal twitch force (*P*_t_) was achieved.

Isometric twitch and tetanic contractile force were assessed by stimulating the muscle for a single twitch then incrementally increasing stimulation frequency (EDL: 10, 40, 80, 120, 150, and 180 Hz; soleus: 10, 40, 80, 100, 120, 150 Hz) with a 1 min rest in between contractions until maximum tetanic force was achieved. Physiological cross-sectional area (PCSA) was calculated by the formula: Muscle mass (g)/[*L* _0_ (cm) * 1.06 (g/cm^3^)] and was subsequently used to calculate specific tetanic force. The half-relaxation time and rate of force development (RFD) at twitch was calculated as described [20]. Fatigability was evaluated by delivering repetitive tetanic stimulations at 80 Hz for both muscles, with 5 s rest in between stimuli and calculating the total area under the force–time curve (AUC). Afterwards, force recovery was measured by delivering ten stimulations at 80 Hz (EDL) or 150 Hz (soleus) at 5 min intervals following fatigue testing. The initial recovery rate (e.g., the slope derived from the end of the fatigue assessment to the first stimulation) and the total force recovered over the period were determined. Muscles that were damaged/torn were excluded from subsequent analyses, but values from previous analyses where the muscle was still viable were included. A flow diagram illustrating the full protocol and muscles included for each contractile assessment is provided in Supplemental Fig. 1.

### RNA extraction

RNA was extracted as previously described [21, 22]. Briefly, one gastrocnemius muscle was pulverized in liquid nitrogen using a chilled pestle and mortar. A ~ 25 mg portion was homogenized in 600 µL of Zymo Tri Reagent (Irvine, CA), and total RNA was isolated using a Zymo RNA Miniprep kit including an on-column DNase treatment (Irvine, CA). RNA quality was determined by the RNA integrity number (RIN; mean: 8.4, range 7.8–8.9) using RNA 6000 Pico Assay RNA chips run in an Agilent 2100 Bioanalyzer (Agilent Technologies, Palo Alto, CA, USA). *n* = 3–4 samples per group were randomly selected and subjected to RNA sequencing.

### Bulk RNA sequencing and gene set enrichment analysis (GSEA)

RNA sequencing was performed by the Microarray and Sequencing Resource Core Facility at Boston University. FASTQ files were aligned to mouse genome build mm10 using STAR (version 2.7.9a) [23]. Ensembl Gene-level counts for non-mitochondrial genes were generated using featureCounts (Subread package, version 1.6.2) and Ensembl annotation build 100 (uniquely aligned proper pairs, same strand). Separately, SAMtools (version 1.10) was used to count reads aligning in proper pairs at least once to either strand of the mitochondrial chromosome (chrM) or to the sense or antisense strands of Ensembl loci of gene biotype “rRNA” or of non-mitochondrial RepeatMasker loci of class “rRNA” (as defined in the RepeatMasker track retrieved from the UCSC Table Browser). FASTQ quality was assessed using FastQC (version 0.11.7), and alignment quality was assessed using RSeQC (version 3.0.0). Variance-stabilizing transformation (VST) was accomplished using the varianceStabilizingTransformation function in the DESeq2 R package (version 1.23.10) [24]. Differential expression was assessed using the Wald test implemented in the DESeq2 R package. Correction for multiple hypothesis testing was accomplished using the Benjamini–Hochberg false discovery rate (FDR). Differentially expressed genes (DEGs) were determined at an FDR q < 0.1. Overrepresentation analyses (ORA) of individual DEG lists were performed by uploading into the Database for Annotation, Visualization, and Integrated Discovery (DAVID) as described [25, 26], and using a modified Fisher’s exact (EASE) score of < 0.05 to determine category enrichment. All analyses were performed using the R environment for statistical computing (version 4.1.2).

Gene Set Enrichment Analysis (GSEA) (version 2.2.1) [27] was used to identify biological terms, pathways and processes that are coordinately up- or down-regulated within each pairwise comparison. The Entrez Gene identifiers of all genes in the Ensembl Gene annotation were ranked by the Wald statistic computed for each coefficient in the two-factor model and for each pairwise comparison. Ensembl Genes matching multiple Entrez Gene IDs were excluded prior to ranking, so that the ranked list represents only those Entrez Gene IDs that match exactly one Ensembl Gene. Each ranked list was then used to perform pre-ranked GSEA analyses (default parameters with random seed 1234) using the Entrez Gene versions of the H (Hallmark), M2 CP (Biocarta, Reactome, WikiPathways), and M5 (Gene Ontology, GO) gene sets obtained from the Molecular Signatures Database (MSigDB), version 2024.1.Mm. An FDR q < 0.05 was used to denote significant enrichment of gene sets for GSEA analysis. Enrichment maps were generated using Cytoscape (version 3.10.3) [28]. Gene sets with FDR q < 0.05 were included as nodes and edge threshold was set at a combined similarity coefficient threshold > 0.375. The resulting network was clustered using the Markov cluster algorithm (MCL) with an inflation parameter of 2. Data from the RNA sequencing analysis are accessible in GEO (accession #GSE301564**)** and as a supplement (Supplemental Tables 1–2).

### Statistical analysis

Two-way ANOVA was used to evaluate differences in muscle contractile parameters using Frailty (Low vs. High groups) and Run (Control vs. Run) as the two factors. When a significant interaction was detected, Sidak’s multiple comparisons test was used post hoc. Because assigning animals to frailty groups inherently results in an unbalanced sample size that reduces power to detect interactions, the percent change from each mouse of the running group to the mean value of its corresponding control group was calculated for each contractile parameter and analyzed using unpaired two-tailed *t*-tests. To assess recovery dynamics, the entire recovery force–time curve was initially analyzed by Three-way ANOVA with Contraction, Frailty, and Run as factors. Where a three-way interaction was observed, follow-up analyses were further stratified by frailty and assessed by separate two-way ANOVAs. Analyses were performed on GraphPad Prism (version 10.5). Significance was set at p < 0.05 and data are presented as mean ± standard deviation. RNA sequencing analysis is described above.

## Results

### Extent of frailty determination

**Fig. 2:**
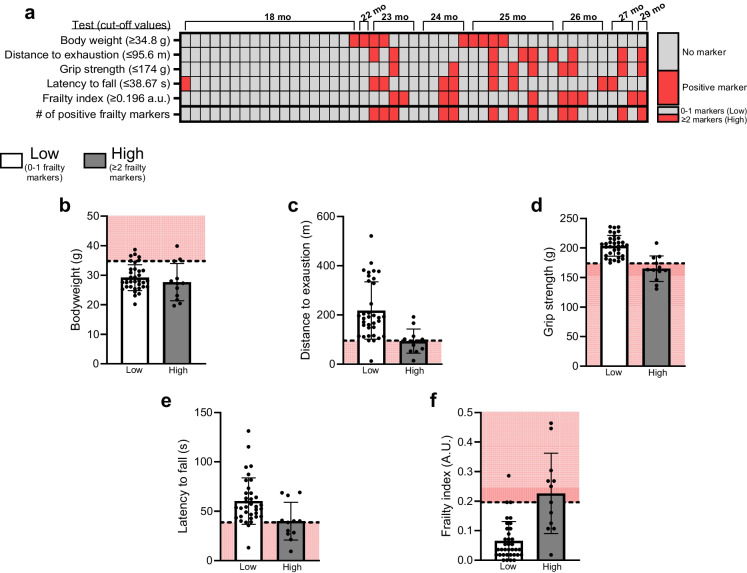
Group allocation by frailty phenotyping. (**a**) Heat map displaying results of frailty phenotyping of aged female mice (*n* = 47) across health and performance tests. Each column represents a single mouse. Mice received a positive frailty marker (shown as red box) if they fell above the 80th percentile of the present cohort values for body weight or frailty index (FI), or below the 20th percentile for treadmill distance to exhaustion, grip strength, or Rotarod latency to fall. Animals were placed in the High group (*n* = 35) if they exhibited ≥ 2 positive markers or Low group (*n* = 12) if they exhibited ≤ 1 frailty markers. Distribution of mouse values for each test following assignment to High or Low groups are visualized in (**b**-**f**). Values falling within the shaded red area in (**b**-**f**) indicates a frail marker for that test. Data are presented as individual values superimposed on mean ± standard deviation

Forty-seven mice were assessed for indices of frailty using treadmill distance to exhaustion, grip strength, rotarod latency to fall, body weight, and FI. Mice received a positive frailty marker if their value on a given test exceeded a defined threshold. Cut-off values were set at the top 80th percentile of the present cohort for body weight and FI, and the bottom 20th percentile for physical tests (e.g., distance to exhaustion, grip strength, and latency to fall). Based on these established criteria, 12 mice were assigned to the High frailty marker group (≥ 2 positive frailty markers) and 35 mice were assigned to the Low frailty marker group (≤ 1 markers; Fig. 2a). The individual distribution of values for each index of frailty can be visualized in Fig. 2b-f.

### EDL and soleus specific tetanic forces are not altered by the extent of frailty or downhill running

**Fig. 3:**
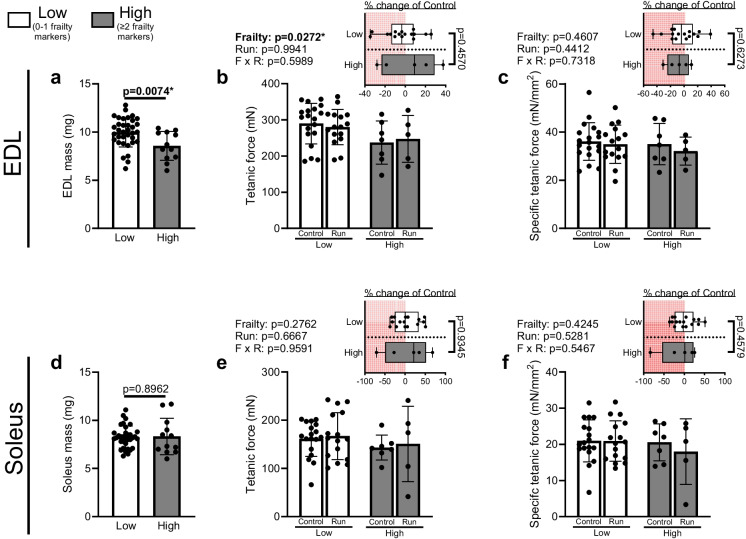
Tetanic and specific tetanic forces generated by the EDL and soleus. (**a**) EDL mass of mice from the High (≥ 2 frailty markers; *n* = 35) or Low (≤ 1 frailty markers; *n* = 12) groups. (**b**) Tetanic force and (**c**) specific tetanic force were assessed in the EDL of sedentary mice from the Low group (Low Control; *n* = 19), ran mice from the Low group (Low Run; *n* = 16), sedentary mice from the High group (Frail Control; *n* = 7), or ran mice from the High group (High Run; *n* = 5). (**d**) Soleus mass of mice from the High (*n* = 35) or Low (*n* = 12) groups. (**e**) Tetanic force and (**f**) specific tetanic force were assessed in the soleus of mice from the Low Control group (*n* = 18), Low Run group (*n* = 16), High Control group (*n* = 7), or High Run group (*n* = 5). (**a**, **d**) were analyzed by unpaired *t*-test. (**b**-**c**, **e**–**f**) were analyzed by two-way ANOVA and the percent change was analyzed by unpaired *t*-test. Data are presented as individual values superimposed on mean ± standard deviation

EDL mass was reduced in High frailty marked mice compared to Low frailty marked mice (Fig. 3a), coinciding with a lower absolute tetanic force output in the High frailty marked groups (Fig. 3b). However, when tetanic force was normalized to the muscle’s physiological cross-sectional area (PCSA), specific force was not different between groups (Fig. 3c). Thus, the lower absolute tetanic force observed in High frailty marked mice was likely driven by the smaller muscle size. In contrast, soleus muscle mass, absolute tetanic force, and specific tetanic force were unaltered by the extent of frailty or the downhill run (Fig. 3d-f).

### Mice with greater frailty indices exhibit enhanced fatiguability and impaired recovery kinetics in the EDL muscle following downhill running

**Fig. 4:**
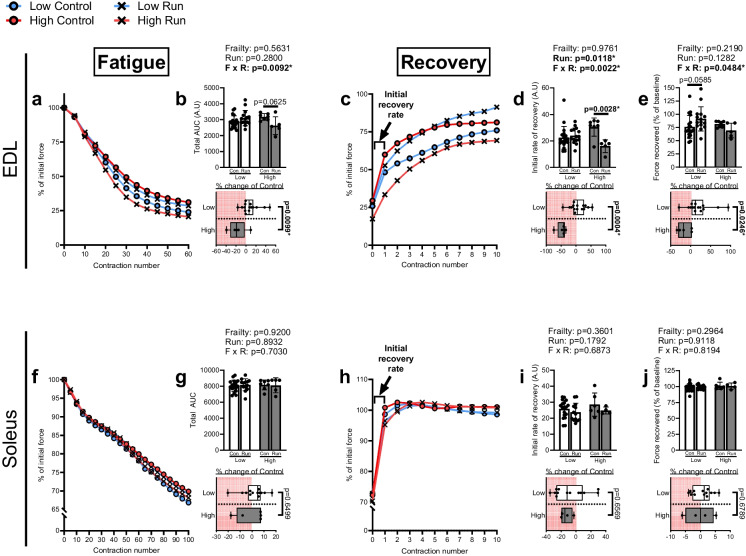
Mice with greater indices of frailty exhibit enhanced fatiguability and impaired recovery kinetics in the EDL following downhill running. (**a**) Representative force–time curve and (**b**) corresponding area under the curve (AUC) following the fatigue protocol in the EDL of sedentary mice from the Low group (≤ 1 frailty markers; Low Control; *n* = 19), ran mice from the Low group (Low Run; *n* = 15), sedentary mice from the High group (≥ 2 frailty markers; High Control; *n* = 7), or ran mice from the High group (High Run; *n* = 5). (**c**) Representative force–time curve, (**d**) initial rate of recovery, and (**e**) the percent force recovered over the recovery protocol from the same EDL muscles as (**b**). (**f**) Representative force–time curve and (**g**) corresponding AUC following the fatigue protocol in the soleus of mice from the Low Control group (*n* = 18), Low Run group (*n* = 13), High Control group (*n* = 7), or High Run group (*n* = 5). (**h**) Representative force–time curve, (**i**) initial rate of recovery, and (**j**) the percent force recovered over the recovery protocol in the soleus from the Low Control group (*n* = 18), Low Run group (*n* = 13), High Control group (*n* = 6), or High Run group (*n* = 4). All data analyzed by two-way ANOVA with percent change analyzed by unpaired *t*-test. Sidak’s multiple comparisons used post hoc in (**b**, **d**-**e**). Data are presented as individual values superimposed on mean ± standard deviation

We next investigated whether downhill running impacted the ability to sustain force output during repetitive contractions (i.e., fatiguability) in mice with varying indices of frailty. Analysis of the total AUC of the EDL force–time curve generated from the fatigue protocol revealed a significant Frailty × Run interaction (Fig. 4a-b). Although post hoc testing only approached a significantly reduced AUC in the run High frailty marked mice compared to the sedentary control High frailty marked mice (*p* = 0.0625), analyzing the percent change between the running groups to their respective controls showed a reduced AUC between High frailty marked mice relative to Low frailty marked mice (Fig. 4b).

We then assessed whether the force recovery kinetics following the fatigue protocol were differentially influenced by running stress between frailty marked groups. To accomplish this, the instantaneous recovery rates between each pair of consecutive contractions were calculated and a three-way ANOVA (Contraction × Frailty × Run) was performed on the resultant derivation plot from all four groups (full statistical analysis is provided in Supplemental Figure 2). A three-way interaction (Contraction × Frailty × Run) was detected, prompting a stratified analysis by frailty status using separate two-way ANOVAs. In doing so, we observed a Contraction × Run interaction solely in the High frailty marked groups, with post hoc testing revealing a significant time point difference only at the initial contraction (Supplemental Figure 2). We therefore focused our analysis on the recovery rate of the initial contraction period (labeled in Fig. 4c, h). Specific analysis of the initial recovery rate in the EDL showed a main effect of running and a Frailty × Run interaction, with post hoc testing revealing an impaired initial rate of recovery in the run High frailty marked group mice relative to their sedentary control counterparts (Fig. 4c-d). We also assessed the total force recovered over the recovery period, expressed relative to the baseline force at the beginning of the fatigue protocol. In the EDL, there was a Frailty × Run interaction detected with post hoc testing approaching a significant effect of greater force recovered in the run Low frailty marked mice relative to their sedentary control counterparts (*p* = 0.0585; Fig. 4c, e). Secondary analysis of the percent change between the running groups to their respective controls confirmed a significantly greater percentage of recovered force in response to running in the Low frailty marked mice relative to High frailty marked mice (Fig. 4e). Repeating these analyses in the soleus revealed no detectable effects of running or extent of frailty on fatiguability or recovery kinetics (Fig. 4f-j).

### Downhill running modifies calcium-sensitive contractile properties of the EDL in both High and Low frailty marked groups

**Fig. 5:**
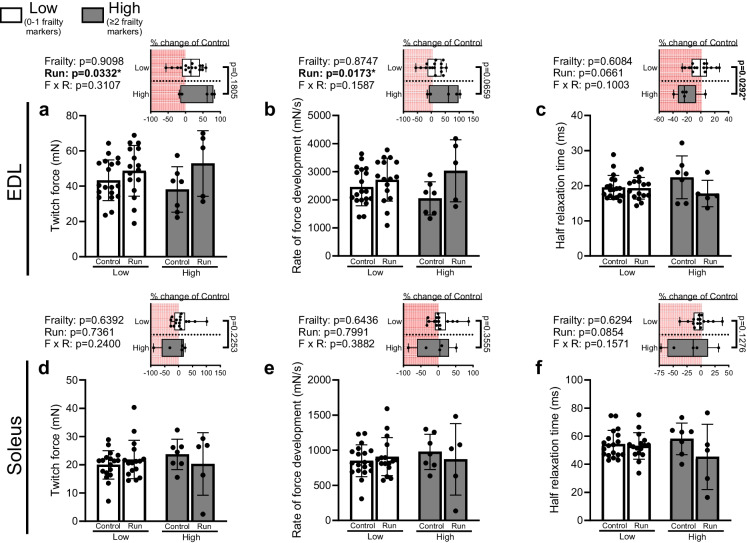
Downhill running modifies calcium-sensitive contractile properties in the EDL. (**a**) Twitch force, (**b**) twitch rate of force development (RFD), and (**c**) twitch half-relaxation time were assessed in the EDL of sedentary mice from the Low group (≤ 1 frailty markers; Low Control; *n* = 19), ran mice from the Low group (Low Run; *n* = 16), sedentary mice from the High group (≥ 2 frailty markers; High Control; *n* = 7), or ran mice from High group (High Run; *n* = 5). (**d**) Twitch force, (**e**) twitch RFD, and (**f**) twitch half-relaxation time were assessed in the soleus of mice from the Low Control group (*n* = 18), Low Run group (*n* = 16), High Control group (*n* = 7), or High Run group (*n* = 5). All data analyzed by two-way ANOVA with percent change analyzed by unpaired *t*-test. Data are presented as individual values superimposed on mean ± standard deviation

Increased muscle fatiguability following repetitive contractions in the absence of altered tetanic force may be partially attributed to altered muscle calcium handling. Thus, we assessed twitch kinetics as a functional indicator of calcium dynamics. In the EDL, downhill running resulted in greater twitch force and RFD, regardless of extent of frailty (Fig. 5a-b). In contrast, half-relaxation time tended to show a reduction in response to downhill running (*p* = 0.0661; Fig. 5c). When analyzing values of running groups as a percent change from their sedentary controls, a greater shortening of half-relaxation time was observed between the High frailty marked mice compared to the Low frailty marked mice (Fig. 5c). These same twitch kinetics were not observed to be altered in the soleus in either High or Low frailty marked groups or in response to running (Fig. 5d-f).

### A divergent transcriptomic signature of mitochondrial-associated genes accompanies changes to the frailty- and running stress-specific changes to muscle contractility

**Fig. 6:**
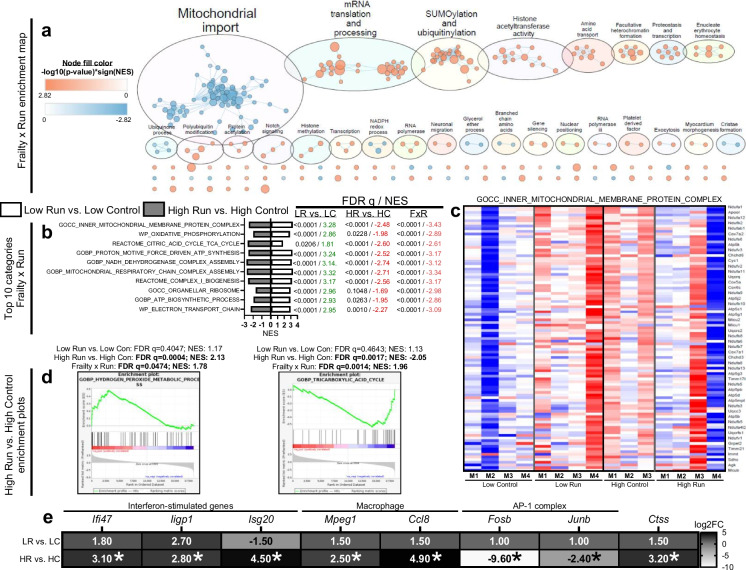
Gene set enrichment analysis (GSEA) on RNA extracted from the gastrocnemius muscle of run mice from the Low (≤ 1 frailty markers; Low Run; *n* = 4) and High groups (≥ 2 frailty markers; High Run; *n* = 4) 24 h post-downhill running compared to their cage sedentary counterparts (Low and High Control; *n* = 3–4). (**a**) Enrichment cluster mapping of enriched gene sets with a Frailty × Run interaction. (**b**) Top 10 enriched GSEA categories with a Frailty × Run interaction by false discovery rate (FDR). LR: Low Run; LC: Low Control; HR: High Run; HC: High Control; FxR: Frailty × Run interaction. NES: Normalized enrichment score. (**c**) Representative leading-edge gene heatmap of select category from the Top 10 enriched GSEA categories. (**d**) GSEA plots of other select significantly enriched gene sets by Frailty × Run interaction. (**e**) Select differentially expressed genes (DEGs) associated with inflammation. Asterisks indicate differential expression at FDR q < 0.1. RNA sequencing data were analyzed by DESeq2

Changes to the skeletal muscle transcriptome are reported 24 h following exercise-induced muscle stress [29, 30]. In the context of advancing chronological age, certain transcriptional responses differ between the muscles of younger and older humans after a bout of eccentric contractions (e.g., inflammatory genes), which have been implicated in muscle resilience [31]. To investigate this in our model, we performed bulk RNA sequencing on the gastrocnemius and focused our analysis on enriched gene programs that were differentially regulated by the running stress in Low and High frailty marked mice. There was a total of *n* = 284 significantly enriched gene sets with a Frailty × Run interaction (FDR q < 0.05). To reveal major biological themes, we organized the enriched gene sets into clusters using network analysis. The network analysis identified several functionally related clusters, with the most highly enriched clusters consisting of gene sets related to mitochondrial import (*n* = 76 clusters), mRNA translation and processing (*n* = 26), and SUMOylation/ubiquitylation (*n* = 24) (Fig. 6a; Supplemental Table 3).

We then evaluated the most highly enriched gene sets by stepwise sorting the GSEA list initially by lowest FDR for the Frailty effect, then subsequently re-sorting that output by lowest FDR for the Frailty × Run interaction. Consistent with our network analysis, the top 10 most significant gene sets (lowest FDR for Frailty × Run interaction) were highly enriched for mitochondrial bioenergetics (e.g., WP_OXIDATIVE PHOSPHORYLATION, GOBP_ATP_BIOSYNTHETIC_PROCESS) and mitochondrial complex assembly processes (e.g., GOBP_NADH_DEHYDROGENASE_COMPLEX_ASSEMBLY, GOCC_MITOCHONDRIAL_RESPIRATORY_CHAIN_COMPLEX_ASSEMBLY; Fig. 6b-c). Intriguingly, the gene set regulation showed clear divergence with respect to High and Low frailty marked groups, whereupon running in Low frailty marked mice led to coordinated upregulation of these gene programs relative to their controls and running in High frailty marked mice led to coordinated downregulation relative to their controls. Additional analysis of significantly enriched gene sets outside of the top 10 most significant supported this divergent transcriptomic signature, with High frailty marked mice exhibiting an exclusive coordinate upregulation of categories related to hydrogen peroxide metabolism (e.g., GOBP_HYDROGEN_PEROXIDE_METABOLIC_PROCESS) and downregulation of categories related to the tricarboxylic acid/Kreb’s cycle (e.g., GOBP_TRICARBOYXLIC_ACID_CYCLE) in response to running that was not observed in Low frailty marked animals (Fig. 6d).

Subsequently, we conducted overrepresentation analyses by pairwise comparison of individual DEG lists between High frailty marked groups (High Run vs. High Control) and Low frailty marked groups (Low Run vs. Low Control). This analysis revealed specific enrichments to inflammation-associated pathways in the High frailty marked groups following running stress. These enrichments included “Innate immune response”, “Phagosome”, “Cellular response to interferon-beta”, “TNF signaling”, “Eosinophil chemotaxis”, and “IL-17 signaling pathway”, none of which observed in the Low group comparison (Supplemental Table 4). Within these categories were running-induced DEGs unique to the High group, including upregulation of interferon stimulated transcripts (*Ifi47, Iigp1, Isg20*), macrophage-associated transcripts (*Mpeg1, Ccl8*), and Cathespin S (*Ctss)*, along with downregulation of components of the inducible inflammation-modulatory Activator protein 1 (AP-1) transcriptional complex (*Fosb, Junb*) (Fig. 6e).

## Discussion

The objective of this work was to define how increasing indices of frailty impacts resilience and resistance to physical stress in aged skeletal muscle. We demonstrate that greater frailty status modifies the muscular response to downhill running stress, whereupon mice with higher indices of frailty show greater fatiguability and dysregulated recovery kinetics 24 h post-stressor compared to their counterparts with lower indices of frailty. These changes occurred despite similar specific isometric tetanic force generation capacity. Downhill running also impacted functional indices of calcium handling in both groups of animals, although modest changes to half-relaxation time occurred exclusively in High frailty marked animals. Contractile alterations occurred specifically in the EDL and not the soleus muscle, potentially indicating a muscle-specific response. Transcriptomic profiling 24 h following downhill running revealed a striking divergence in mitochondrial gene regulation between High and Low frailty marked mice in response to running. Namely, while Low frailty marked mice exhibited coordinated upregulation of gene programs associated with mitochondrial bioenergetics and complex assembly, High frailty marked mice showed a downregulation of these same pathways. Our collective data more thoroughly defines the impact of frailty extent on resilience and resistance to stress in skeletal muscle. Additionally, these data suggest a potential role for altered mitochondrial function in the frailty-associated changes to resilience and resistance following the physiological stress of downhill running.

Although both High and Low frailty marked mice maintained specific tetanic force production following downhill running, the EDL of High frailty marked mice exhibited greater fatiguability after repetitive stimulation. While increasing chronological age is associated with losses to maximum force generation [32], our data imply that decreased maximal force during frailty is largely driven by decreased muscle size and not necessarily reduced intrinsic capacity of the muscle contractile machinery (e.g., actin and myosin) per se. Notably, the reduced muscle size in High frailty marked mice compared to Low frailty marked mice was specific to the EDL, aligning with the greater susceptibility to selective atrophy and loss of type II fibers with advancing age [33, 34]. Our findings instead suggest latent impairments in stress resilience or resistance in frail muscle, whereby exposure to physical stress reduces the muscle’s ability to sustain and recover force following repeated contractions. The dissociation between maximum force generation and fatiguability suggests that factors such as calcium handling, redox balance, and/or mitochondrial function may contribute to these deficits observed with frailty following downhill running.

We observed shortening of half-relaxation time following the running stress in the High frailty marked mice, despite both groups exhibiting greater twitch force and RFD post-running. One possible mechanism underlying these calcium-sensitive properties is alterations to redox balance. Reactive oxygen species (ROS) are generated during exercise stress [35] and are produced by immune cells responding to any subsequent muscle damage [36]. Paradoxically, skeletal muscle ROS have been observed to both increase or decrease twitch contractile properties/calcium kinetics in part by oxidatively modifying sarcoplasmic reticulum or myofibrillar regulatory proteins [37–39]. A proposed model is that ROS act hormetically, whereby low-to-moderate amounts of ROS enhance calcium handling and twitch contractile properties while excessive amounts inhibit muscle function [40]. In contrast, studies investigating the impact of ROS generation on muscular fatigue more consistently demonstrate that ROS accumulation contributes to fatiguability during repetitive contractions [41]. It is plausible that ROS were generated by both High and Low frailty marked mice during downhill running that resulted in improved calcium release and twitch kinetics 24 h later, possibly via oxidative modifications to proteins such as the ryanodine receptor (RyR) [42]. However, differences in the magnitude or duration of ROS exposure in High frailty marked mice, either during the run or the subsequent regenerative period, may have contributed to greater fatiguability and shortened relaxation time. To illustrate, ex vivo preparations have shown that ROS can alter Ca^2^⁺-myosin cooperativity via troponin C (TnC) [38] and inhibit SERCA-mediated calcium reuptake [37, 39]. Theoretically, disrupted cooperativity at the myofibrillar apparatus could reduce Ca^2^⁺-myosin binding (e.g., promoting Ca^2^⁺ dissociation and thereby accelerating time to relaxation) whereas simultaneous SERCA inhibition could impair subsequent reuptake of the dissociated cytoplasmic Ca^2^⁺, compromising calcium cycling efficiency required for repetitive contractions and thus contributing to fatigue.

There is a substantial interplay between calcium availability and mitochondrial function. It is possible that the increased calcium availability proposed to influence enhanced twitch kinetics after downhill running would likely impose additional buffering demands on the mitochondria. Although moderate concentrations of calcium at the mitochondria enhance ATP synthesis, calcium overload reduces ATP synthesis and induces mitochondrial stress [43, 44]. Aging is associated with diminished mitochondrial resilience [45], and our transcriptomic profiling revealed a divergent mitochondrial gene expression response to running between mice with High and Low frailty markers, suggesting a differing mitochondrial burden. Mitochondrial oxidative metabolism provides a source of ROS during exercise [46], and dysfunctional mitochondria may overproduce ROS [47]. Consistent with this, our gene enrichment analysis revealed significant upregulation of hydrogen peroxide metabolism pathways exclusively in High frailty marked mice following running. Moreover, muscles composed predominantly of type II fibers (such as the EDL) tend to generate more mitochondrial ROS compared to type I fiber-rich muscles like the soleus [48], aligning with our functional observations being specific to the EDL. Collectively, these findings support the interpretation that the greater fatiguability and reduced half-relaxation time observed in High frailty marked mice after downhill running may be at least partially attributable to disrupted mitochondrial and/or redox regulation.

We also observed distinct effects of downhill running on muscle recovery kinetics between High and Low frailty marked mice. The initial rate of recovery was reduced in run High frailty marked mice relative to their controls. Phosphocreatine (PCr) resynthesis is a key determinant of acute force recovery after repeated contractions, and PCr recovery is prolonged with advancing age [49]. Since PCr recovery is dependent on ATP availability, mitochondrial dysfunction can impair this process and remains a likely mechanism [49, 50]. In contrast, run Low frailty marked mice exhibited greater force recovery, aligning with reports that a single extended exercise bout can rapidly improve ATP and PCr maintenance relative to their breakdown products (e.g., ADP/AMP, Cr/P_i_) [51]. This adaptation towards a more protected energy state is at least partially dependent on intact mitochondrial function [52]. The impaired recovery in run High frailty marked group mice may also reflect aberrant inflammation. Chronological aging is associated with heightened and/or prolonged exercise-induced inflammatory responses that impede repair and adaptation [53, 54]. Consistent with this, our RNA sequencing overrepresentation analysis revealed upregulation of transcripts such as interferon-stimulated genes (e.g., *Ifi47, Iigp1,* and *Isg20*) and macrophage-produced genes (e.g., *Mpeg1* and *Ccl8*) in response to running in the High frailty marked but not Low frailty marked groups. These findings suggest an increased production and/or reduced resolution capacity of inflammation in run High frailty marked mice. Such aberrant inflammatory responses can exacerbate mitochondrial dysfunction and/or divert ATP away from contractile recovery toward sustaining the inflammatory program [55, 56], potentially compounding any energetic deficits and impairing muscle recovery.

Notably, the alterations to contractile performance were confined to the EDL and were absent in the soleus. Single muscle fiber proteomics from mice and young humans have identified fiber type-specific enrichment patterns of structural, metabolic, and contractile proteins in fibers defined by myosin heavy chain isoform [55, 56]. These proteomic profiles are differentially affected by chronological aging. For example, the contents of carbohydrate metabolism regulatory proteins and actin-myosin chaperones are upregulated in slow fibers but downregulated in fast fibers with age [57]. Thus, potential interaction(s) between frailty severity the distinct fiber type of the EDL and soleus should not be discounted as contributors to the observed contractile phenotypes.

Overall, these findings offer important implications for understanding the physiological underpinnings of frailty-associated muscle dysfunction. Although increasing chronological age is often characterized by diminishing baseline functionality (such as lower absolute force generation in muscle), our data indicates that impaired stress responses are a defining and functionally relevant feature of frailty.. Thus, it is imperative to evaluate frailty under conditions of stress, as impairments may remain latent at basal levels and only present when the system is challenged. For instance, our model uncovered divergent transcriptional responses between mice marked with High or Low frailty indices, suggesting altered redox regulation and mitochondrial function as candidate mechanisms contributing to the reduced muscular resilience to a running stress. These insights highlight potential molecular targets for therapeutic strategies to improve function in frail populations.

There are limitations to consider. First, all assessments were performed in female mice and sex-specific responses may yield differing results in males. Second, our measures were limited to a single post-stress time point (24 h), which may not capture early transcriptional and functional responses in frail mice. Third, while we evaluated three muscles (EDL, soleus, and gastrocnemius) that are mechanically stimulated by downhill running, our findings may not generalize to all lower limb muscles or muscle groups. Lastly, assessments of muscle resilience were limited to a single stressor. Evaluation of other physiological stressors in the context of frailty is warranted in future investigations.

In summary, this work defines key impairments in muscle resistance and resilience to physical stress in the context of frailty. Despite preserved tetanic force, frail animals exhibited greater fatiguability, dysregulated recovery kinetics, and unique transcriptomic responses suggestive of altered mitochondrial and inflammatory signaling. These responses were specific to the EDL and were not observed in the soleus, implying the potential of fiber type-specific changes. Future work should employ directed gain/loss-of-function approaches to assess the contribution of these pathways and identify the likelihood of fiber type-specific regulation. These findings reinforce that frailty is not merely a reflection of chronological age or muscle mass, but rather a state of impaired resilience and resistance to stress.

## Supplementary Information

Below is the link to the electronic supplementary material.

Supplementary file1 (PDF 158 KB)

Supplementary file2 (PDF 30 KB)

Supplementary file3 (PDF 16 KB)

Supplementary file4 (CSV 223 KB)

Supplementary file5 (XLSX 14 KB)

## Data Availability

RNA sequencing data are accessible in GEO (accession #GSE301564). All other raw data will be made available by reasonable request.
