# Supplementary material for: Increasing indices of frailty in aged female mice are associated with impaired skeletal muscle resilience to downhill running stress
Source: GeroScience. 2025 Sep 11;48(2):1665–81. doi: 10.1007/s11357-025-01856-7 (PMC12972430; doi:10.1007/s11357-025-01856-7)
Supplement: Supplementary file 2 — Supplementary file2 (PDF 30 KB) [file 11357_2025_1856_MOESM2_ESM.pdf]

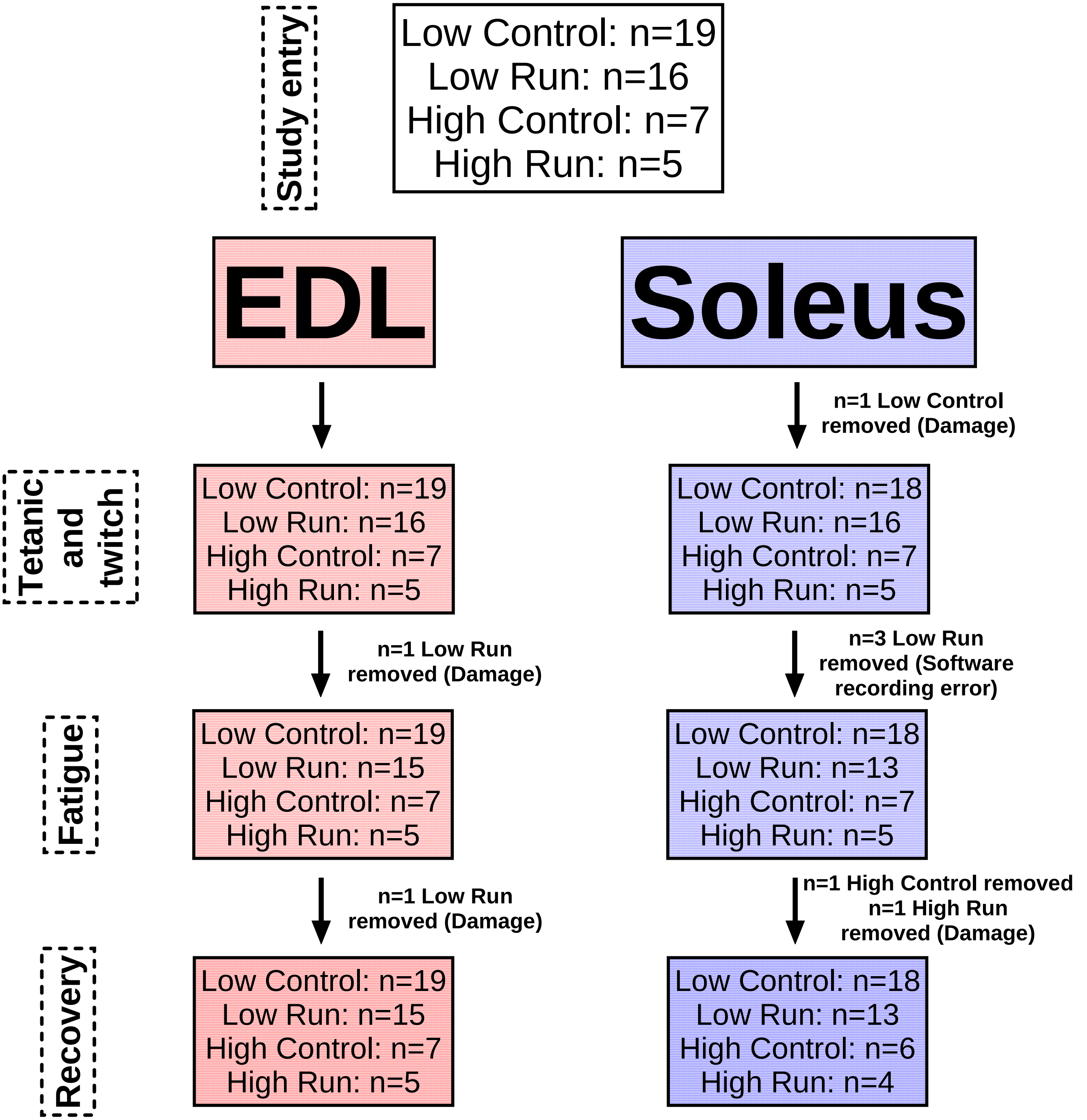

Supplemental Fig. 1: Flow diagram outlining the sequence of *ex vivo* muscle physiology used to assess EDL and soleus muscles from mice stratified to High (≥2 frailty markers) or Low (≤1 frailty markers) groups that were subjected to running (Run) or remained cage sedentary (Control). Only muscles that were viable (not damaged) were used for subsequent analysis.
