# Supplementary material for: Increasing indices of frailty in aged female mice are associated with impaired skeletal muscle resilience to downhill running stress
Source: GeroScience. 2025 Sep 11;48(2):1665–81. doi: 10.1007/s11357-025-01856-7 (PMC12972430; doi:10.1007/s11357-025-01856-7)
Supplement: Supplementary file 3 — Supplementary file3 (PDF 16 KB) [file 11357_2025_1856_MOESM3_ESM.pdf]

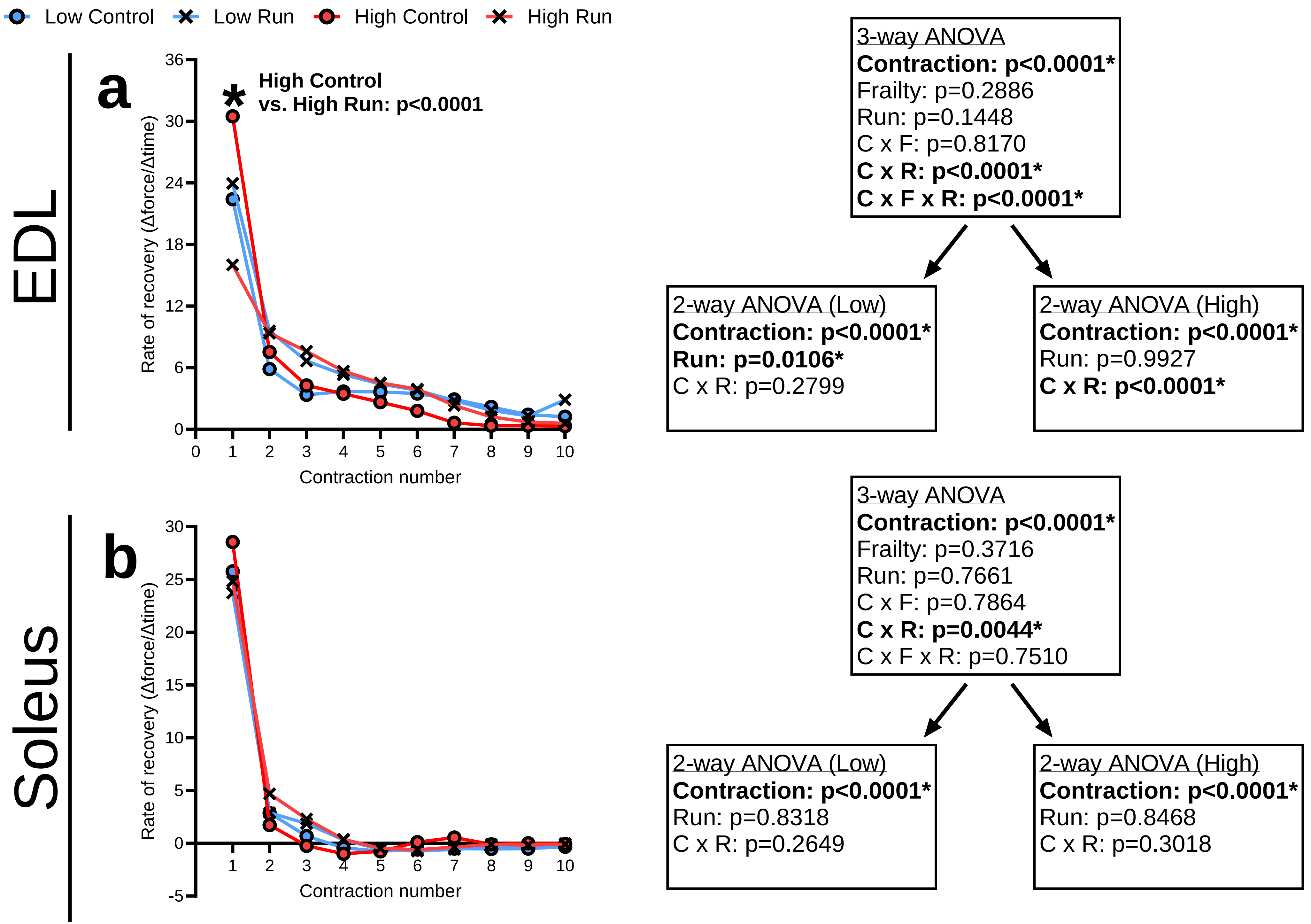

Supplemental Fig. 2: Full statistical analysis of rate of recovery in (A) EDL and (B) soleus muscles. The instananeous rate of recovery was derived between each pair of consecutive contractions and a three-way ANOVA (Contraction × Frailty × Run) was performed on the resultant derivation plot from all four groups. Stratified two-way ANOVAs by Frailty were performed where a 3-way interaction was detected in (A). Data still shown for the stratified analysis in (B). Contraction × Run interaction was detected in EDL muscle between the High fraily marked groups. Sidak's multiple comparisons test post hoc revealed significant time point difference only at the initial contraction.
